# An Improved Calibration Method for the IMU Biases Utilizing KF-Based AdaGrad Algorithm

**DOI:** 10.3390/s21155055

**Published:** 2021-07-26

**Authors:** Zeyang Wen, Gongliu Yang, Qingzhong Cai

**Affiliations:** School of Instrumentation and Optoelectronic Engineering, Beihang University, Beijing 100191, China; cruyffwen@163.com (Z.W.); yanggongliu@buaa.edu.cn (G.Y.)

**Keywords:** inertial measurement unit (IMU) calibration, strapdown inertial navigation system (SINS), Kalman filter, gradient descent

## Abstract

In the field of high accuracy strapdown inertial navigation system (SINS), the inertial measurement unit (IMU) biases can severely affect the navigation accuracy. Traditionally we use Kalman filter (KF) to estimate those biases. However, KF is an unbiased estimation method based on the assumption of Gaussian white noise (GWN) while IMU sensors noise is irregular. Kalman filtering will no longer be accurate when the sensor’s noise is irregular. In order to obtain the optimal solution of the IMU biases, this paper proposes a novel method for the calibration of IMU biases utilizing the KF-based AdaGrad algorithm to solve this problem. Three improvements were made as the following: (1) The adaptive subgradient method (AdaGrad) is proposed to overcome the difficulty of setting step size. (2) A KF-based AdaGrad numerical function is derived and (3) a KF-based AdaGrad calibration algorithm is proposed in this paper. Experimental results show that the method proposed in this paper can effectively improve the accuracy of IMU biases in both static tests and car-mounted field tests.

## 1. Introduction

Strapdown Inertial Navigation Systems (SINS) have been widely used in many aspects of navigation [1]. The SINS has some irreplaceable advantages, such as high autonomy, and it provides continuous and comprehensive navigation information. Therefore, SINS is widely applied in ships and airplanes [2]. For the purpose of realizing the alignment and navigation algorithms of the SINS. The IMU errors have to be calibrated prior to utilization. In an ideal IMU, accelerometers, and gyroscopes coordinate with the same orthogonal sensitivity axes, while the scale factor converts the digital quantity measured by each sensor into the real physical quantity (accelerations and angular rates) [3,4]. However, the IMU is usually affected by non-accurate sensor biases [5], the navigation errors (attitude, velocity and position errors) of the SINS diverge with time [6]. Therefore, the calibration of biases in IMU is a very effective way to enhance the navigation accuracy of SINS.

In [7], Han et al. proposed a method for bias calibration using a single-axis turning table. The disadvantage of this method is that the calibration accuracy depends highly on the turntable accuracy. In current SINS implementations, in order to overcome the non-observability of the biases of the IMU biases, an analytic calibration of IMU biases with two-position is utilized widely [8]. Many works have been done in the field of IMU calibration. In [9], an eight-position self-calibration method was proposed. Emel’yantsev et al. calibrated in-rum drifts of SINS with uniaxial modulation rotation of measurement unit. Since the low end IMU model is nonlinear, some researchers use an adaptive Kalman filter to estimate IMU biases [10,11,12,13,14,15]. Wang et al. utilized a new online calibration method for integrated navigation systems [16]. In [17], a rotation test was utilized to enhance the observability of KF in order to calibrate the IMU biases. However, KF is an unbiased estimation method based on the assumption of GWN while IMU sensors noise is irregular [18,19,20]. In [21], an autocovariance least-squares (ALS) technique was proposed to estimate the process noise of IMU. In [22], particle swarm method was utilized in gyro drift estimation. For high-dimensional systems especially for those with weak observability. The adaptive estimation of IMU biases tend to diverge. In order to solve the problem, further researches are needed on this issue.

Allan variance has been widely utilized in the analysis of gyroscopes [23]. Allan variance can provide different types and magnitude information on various noise terms. Due to analogies to IMU sensors, Allan variance has been adapted to the random-drift characterization of a variety of inertial devices [24,25,26]. Allan variance is a method of representing the root mean square (RMS) random-drift errors as a function of averaging time. However, the equivalent white noise characteristics do not equal the sum of the various noise characteristics analyzed by Allan variance. Thus, the method of using Allan variance to estimate IMU biases is not feasible.

In order to solve the above problems, a KF-based AdaGrad calibration algorithm is proposed. Gradient descent is an iterative optimization algorithm for finding the minimum value of the objective function. For a complex dynamical system, the gradient descent method is utilized to obtain the minimum value of the constructed cost function. In [27,28,29,30], researchers proposed a gradient descent approach to solve complex dynamic system problems. The difficulty of the gradient descent method lies in constructing the cost function of a complex dynamic system. In this paper, (1) in order to solve the problem of setting step size, this paper utilized adaptive subgradient methods (AdaGrad); (2) we derive a KF-based AdaGrad numerical cost function in which the objective function is the velocity and position errors; and (3) utilizing the above two theories we propose a novel method called KF-based AdaGrad algorithm. Finally, static tests and field tests are carried out to verify the feasibility and applicability of the investigated method.

The remainder of this paper is organized as follows. Section 2 presents a brief overview of the calibration process of IMU and an analysis of the observability of IMU biases. In Section 3, we derive a KF-based AdaGrad numerical cost function and propose a KF-based AdaGrad calibration algorithm. Rotation tests and car-mounted field tests are carried out in Section 4 to compare the proposed method and the existing calibration method. The conclusions are given in Section 5.

## 2. Two-Position Calibration Modeling

The navigation performance of the SINS relies on a high-precision IMU. Error models have been developed for the IMU in [7,8]. Under the condition of the static base, the velocity of the navigation solution is the velocity error. According to the SINS error transformation model, the misalignment angle error can be derived from the velocity error. Since the inertial navigation system has no obvious movement of the geographical position, the velocity of the inertial navigation system approaches zero. Therefore, in the calibration process, the SINS navigation algorithm can be replaced by some simplified equations.

In a SINS, the IMU is directly mounted on the vehicle or the carrier without a stable platform. The angular velocity and acceleration in the body frame (denoted by *b*) are measured by the gyroscopes and accelerometers. Choose the local geographical frame (East-North-Vertical frame) as the navigation frame (denote by *n*).

Let $\omega_{en}=0$ to get the simplified attitude algorithm:

(1) $${\dot{C}}_{b}^{n}=C_{b}^{n}\left[(\omega_{ib}^{b}-\omega_{ie}^{b})\times \right]$$

$C_{b}^{n}$ is the direction cosine matrix to transform vector from *b*-frame (IMU body orthogonal frame which aligned with Right-North-Up axes) to *n*-frame (navigation frame which aligned with East-North-Vertical axes). The subscript *i* denotes the inertial-frame. $\omega_{ib}^{b}$ represents the angular velocity measured by the gyroscope in the *b*-frame. $\omega_{ie}^{b}$ is the self-rotation angular velocity.

Specific force equation as one of the basic equations of the SINS can be written as:

(2) $${\dot{v}}^{n}=C_{b}^{n}f_{sf}^{b}-\left(2\omega_{ie}^{n}+\omega_{en}^{n}\right)\times v^{n}+g^{n}$$

where $v^{n}={\left[\begin{array}{ccc} v_{E}^{n} & v_{N}^{n} & v_{U}^{n} \end{array}\right]}^{T}$, *g* is the gravity acceleration of the earth. Let $v^{n}=0$ to obtain the simplified velocity algorithm:

(3) $${\dot{v}}^{n}=C_{b}^{n}f_{sf}^{b}+g^{n}$$

Following the above derivation, the attitude error equation and the velocity error equation can be written as:

(4) $$\begin{array}{c} \dot{\phi }=\phi \times \omega_{ie}^{n}-\varepsilon^{n}\\ \delta {\dot{v}}^{n}=f_{sf}^{n}\times \phi +\nabla^{n} \end{array}$$

where ϕ is the misalignment angle vector and $\delta v^{n}$ is the velocity error vector. $\varepsilon^{n}$ is the gyroscope equivalent constant bias and the $\nabla^{n}$ is the accelerometer equivalent constant bias. The expressions for $\varepsilon^{n}$ and $\nabla^{n}$ are written as follows:

(5) $$\varepsilon^{n}=\left[\begin{array}{c} \varepsilon_{E}\\ \varepsilon_{N}\\ \varepsilon_{U} \end{array}\right]=\left[\begin{array}{c} C_{11}\varepsilon_{x}^{b}+C_{12}\varepsilon_{y}^{b}+C_{13}\varepsilon_{z}^{b}\\ C_{21}\varepsilon_{x}^{b}+C_{22}\varepsilon_{y}^{b}+C_{23}\varepsilon_{z}^{b}\\ C_{31}\varepsilon_{x}^{b}+C_{32}\varepsilon_{y}^{b}+C_{33}\varepsilon_{z}^{b} \end{array}\right]$$

(6) $$\nabla^{n}=\left[\begin{array}{c} \nabla_{E}\\ \nabla_{N}\\ \nabla_{U} \end{array}\right]=\left[\begin{array}{c} C_{11}\nabla_{x}^{b}+C_{12}\nabla_{y}^{b}+C_{13}\nabla_{z}^{b}\\ C_{21}\nabla_{x}^{b}+C_{22}\nabla_{y}^{b}+C_{23}\nabla_{z}^{b}\\ C_{31}\nabla_{x}^{b}+C_{32}\nabla_{y}^{b}+C_{33}\nabla_{z}^{b} \end{array}\right]$$

where $C_{ij}$ denotes the elements of $C_{b}^{n}$. $\varepsilon^{b}$ and $\nabla^{b}$ are the gyroscope and accelerometer equivalent constant bias.

In the above equation, there is no cross-linking relationship between the Equation ($\delta {\dot{v}}_{U}=\nabla_{U}$) and the other equations. It can be concluded that the vertical velocity error does not have any effect on the misalignment angle estimation. Therefore, in the analysis of the misalignment angle estimation, the influence of the vertical velocity and vertical accelerometer bias can generally be ignored. By expanding the $\varepsilon^{b}$ and $\nabla^{b}$ to the state, the calibration state space model can be established as follows:

(7) $$\left\{\begin{array}{c} \dot{X}=\mathrm{FX}+GW^{b}\\ Z=\mathrm{HX}+V \end{array}\right.$$

where

(8) $$F=\left[\begin{array}{c} \begin{array}{cccc} -(\omega_{ie}^{n}\times ) & 0_{3\times 3} & -C_{b}^{n} & 0_{3\times 3}\\ -(g^{n}\times ) & 0_{3\times 3} & 0_{3\times 3} & C_{b}^{n} \end{array}\\ 0_{6\times 12} \end{array}\right]$$

(9) $$G=\left[\begin{array}{c} \begin{array}{cc} -C_{b}^{n} & 0_{3\times 3}\\ 0_{3\times 3} & C_{b}^{n} \end{array}\\ 0_{6\times 6} \end{array}\right]$$

(10) $$H=\left[\begin{array}{ccc} 0_{3\times 3} & I_{3\times 3} & 0_{3\times 6} \end{array}\right]$$

According to the designed two-position calibration scheme, the actual system is time-varying system. PWCS method and SVD method are both employed for the two-position calibration scheme. The rank of this observability matrix is 7 [26,27]. Five states $\nabla_{E}$, $\nabla_{N}$, $\varepsilon_{E}$, $\varepsilon_{N}$ and $\varepsilon_{U}$ are considered unobservable. In order to improve the observability, two-position calibration is needed. This is equivalent to changing the $C_{b}^{n}$ of the SINS. Furthermore, changing the SINS error equation from a stationary system to a time-varying system, which is beneficial to improve the observability of the IMU biases. The scale factor errors of the three gyros and accelerometers are not observable during the process of two-position calibration. Therefore, we do not consider the scale factor errors in Equation (7).

Using the piece-wise constant system (PWCS) observability analysis method [27], the rank of the observability matrix is 11 except $\varepsilon_{U}$.

The value of the elements of the process noise matrix [29,30,31], which is the directly determined by the value of the elements in the ***W*** matrix. As is shown in Equation (12), the covariance of the ***W*** matrix is ***Q***. Furthermore, the accuracy of the KF is determined by the reliability of the ***Q*** information. ***W*** and ***Q*** can be written as:

(11) $$W^{b}={\left[\begin{array}{cccccc} w_{gx}^{b} & w_{gy}^{b} & w_{gz}^{b} & w_{ax}^{b} & w_{ay}^{b} & w_{az}^{b} \end{array}\right]}^{T}$$

(12) $$\left\{\begin{array}{c} E\left[W_{k}\right]=0,E\left[W_{k}W_{j}^{T}\right]=Q_{k}\delta_{kj}\\ E\left[V_{k}\right]=0,E\left[V_{k}V_{j}^{T}\right]=R_{k}\delta_{kj}\\ E[W_{k}V_{j}^{T}]=0 \end{array}\right.$$

However, KF is an unbiased estimation method based on the assumption of GWN while IMU sensor noise is irregular. In order to obtain the optimal solution of IMU biases, this paper presents a novel method for the calibration of IMU biases utilizing the KF-based AdaGrad algorithm in Section 4.

## 3. KF-Based AdaGrad Algorithm

### 3.1. Gradient Descent Algorithm

Gradient descent is an iterative algorithm to minimize an objective function. If the multi-variable function F(x) is defined and differentiable. Given an initial point, and then follow the negative of the gradient of the function at every iteration. When the point moves to a critical point where the point does not change anymore (or change a value that approaches zero), it has reached the desired local minimum. The basic algorithm is written as follow:

(13) $$x_{n+1}=x_{n}-\gamma_{n}\nabla F\left(x_{n}\right)$$

where x is the variable vector and γ is the step size.

However, setting the step size of gradient descent is a key issue. An inappropriate step size may cause the function F(x) easily trapped in saddle points. In this paper, the sampling rate of the IMU is 200 Hz. Therefore the sampling time is 0.005 ms. In order to ensure that there are no large values at the beginning. The step size we set is 0.005 at the beginning.

In order to solve this problem, we utilized two methods: line search and adaptive subgradient methods (AdaGrad).

### 3.2. Line Search

Line search is an approach is to adapt the step size at every iteration. The line search will calculate the descent direction $p_{k}$ and the step size $\alpha_{k}$ to move in this direction. The following condition must be satisfied: ${p_{k}}^{T}\nabla F_{k}<0$ so that the function F(x) can be guaranteed to fall along this direction. Furthermore, the search direction can be written as: $p_{k}=-B_{k}^{-1}\nabla F_{k}$. The step size $\alpha_{k}$ should minimize the following function:

(14) $$\phi \left(\alpha \right)=F\left(x_{k}+\alpha p_{k}\right)$$

However, it is difficult to obtain the α that minimizes the above equation, and the amount of calculation is relatively large. The commonly used method is to obtain a larger step size as much as possible with an acceptable amount of calculation, in order to reduce the value of ϕ(α) as much as possible. The general line search method consists of the following two steps:

1. Bracketing: Finding a range containing ideal steps.
2. Dichotomy or Interpolation: Using the dichotomy or interpolation to find the step size in this interval.

The KF-based AdaGrad algorithm is very computationally intensive. Even though the above method can reduce a lot of calculations, a faster step-size adaptive approach is needed. Thus, we utilized AdaGrad to solve this problem.

### 3.3. Adaptive Subgradient Methods (AdaGrad)

The basic idea of AdaGrad is to use different step sizes at every iteration. The step size is large at the beginning for rapid gradient descent. As the optimization process progresses, the step size is reduced for the gradient that has fallen significantly, and the larger step size is maintained for the gradient that has not decreased so much.

For the basic Gradient descent, the vectorized algorithm can be written as:

(15) $$x_{k+1}=x_{k}-\eta \nabla F\left(x_{k}\right)$$

AdaGrad multiplies the step size by a parameter that varies with the number of iterations:

(16) $$\begin{array}{c} x_{k+1}=x_{k}-\frac{\eta }{\sqrt{G_{k}+\varepsilon }}\nabla F\left(x_{k}\right)\\ G_{k}=G_{k-1}+\nabla F\left(x_{k}\right) \end{array}$$

where η is a small value to prevent the denominator to be zero.

It is easy to discover that as the algorithm continues to iterate, $G_{k}$ will grow larger, and the step size will grow smaller. The AdaGrad algorithm starts with the convergence of high speed and ends with a small step size.

**Figure 1:**
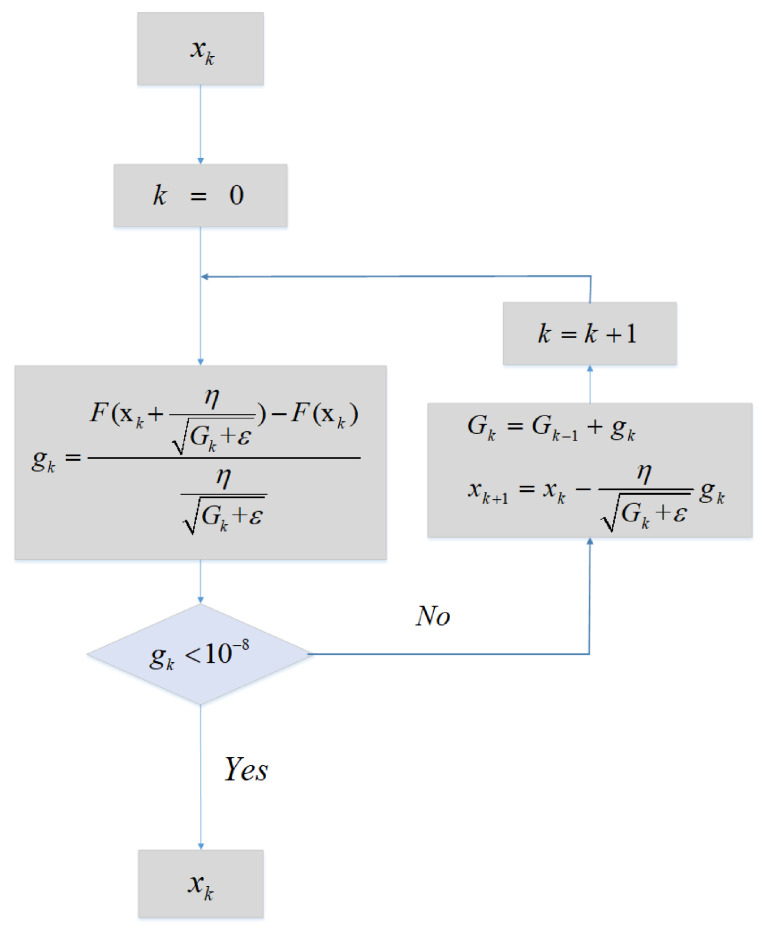
Flowchart of AdaGrad.

Since we proposed a KF-based AdaGrad numerical cost function F(x), the AdaGrad algorithm needs to be discretized. The Algorithm flowchart is shown in Figure 1.

Given $x_{k}$ as an initial value and then enters the loop. When the gradient value $g_{k}$ is close to zero (this paper selects 10e-8 as the critical value). The function jumps out of the loop and the output is $x_{k}$. In this paper, $x_{k}={\left[\begin{array}{cccccc} \varepsilon_{x} & \varepsilon_{y} & \varepsilon_{z} & \nabla_{x} & \nabla_{y} & \nabla_{z} \end{array}\right]}^{T}$, where $Q_{k}$ is the process Noise Covariance Matrix. $F(x_{k})$ is the KF-based numerical function, it is the function which consists of two database and multiple iterations of one KF, we will derive it in the next subsection.

The psudocode is listed in the Algorithm 1 and is shown as follows:
**Algorithm 1:** AdaGrad algorithm.**Input:** The state vector before AdaGrad algorithm.**Output:** The estimated state vector. Initialization:1: $x_{0}={\left[\begin{array}{cccccc} \varepsilon_{x} & \varepsilon_{y} & \varepsilon_{z} & \nabla_{x} & \nabla_{y} & \nabla_{z} \end{array}\right]}^{T}$, k=0, $G_{0}=0$, $g_{k}=0$2: **repeat**:Calculate $g_{k}$:3: $\sqrt{G_{k}+\varepsilon }\eta^{-1}\left[F(x_{k}+\frac{\eta }{\sqrt{G_{k}+\varepsilon }})-F\left(x_{k}\right)\right]$Update $G_{k}$: 4: $G_{k}=G_{k-1}+g_{k}$Update $x_{k}$5: $x_{k}=x_{k-1}-\frac{\eta }{\sqrt{G_{k}+\varepsilon }}g_{k}$6: **until** $g_{k}<1e-8$

### 3.4. KF-Based AdaGrad Calibration Algorithm

AdaGrad is a good method to obtain the minimum value of a function. Setting the IMU biases accurately could improve the accuracy of SINS. In order to obtain the optimal solution of IMU biases, we derives a numerical data function $F(x_{k})$. As $F(x_{k})$ reaches its minimum value, the IMU reaches its biases optimal solution.

In practical utilization, the effective compensation method of IMU biases is conducted as the following steps:

1. SINS (contains IMU and navigation micro-computer) stiffly fixed on a static platform, power up and start navigation.
2. Since the real velocity is 0 under the static platform condition, the pure inertial navigation output velocity is the velocity error. According to the principle of inertial navigation, the velocity error curve will show the form of Schuler oscillation.
3. In the oscillation process of the velocity error curve, the velocity error and positioning error shows the effect of calibration.

As is shown in Algorithm 1, the estimation of $x_{k}$ can be written as:

$$x_{k}=x_{k-1}-\frac{\eta }{\sqrt{G_{k}+\varepsilon }}g_{k}$$

**Figure 2:**
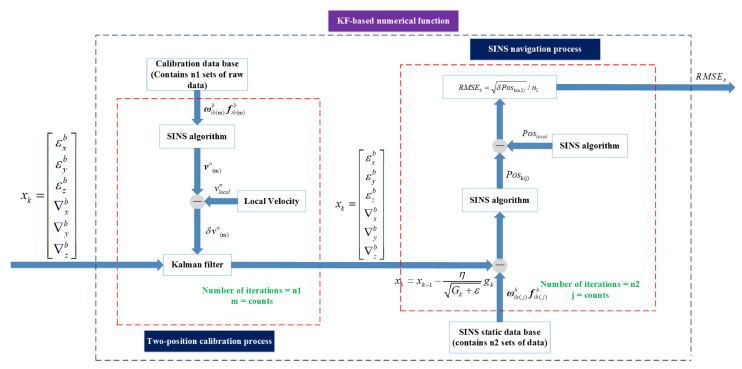
KF-based numerical function diagram.

In summary, if the minimum RMSE of the position error can be obtained, and then the bias compensation effect reaches its optimal. Utilizing a two-position calibration algorithm, IMU biases can be obtained when the process noise matrix is determined. We have derived the KF-based AdaGrad numerical function $F(x_{k})$ and show the diagram of the numerical function in Figure 2. The pseudocode of the KF-based numerical function is listed in Algorithm 2 and is shown as follows.

As is shown in Figure 2, the entire KF-based numerical function $F(x_{k})$ can be divided into two major processes: (1) two-position calibration process and (2) SINS navigation process. A detailed explanation of these two processes is as follows:

1. A two-position calibration process: based on the calibration database (contains n1 sets of data), $\omega_{\mathrm{ib}(m)}^{b}$ and $f_{\mathrm{ib}(m)}^{b}$ can be used for SINS algorithm. According to the theory of Section 3, set IMU biases and using KF to estimate attitude, velocity and position. During every iteration, intermediate variables $\varepsilon_{k(m)}$ and $\nabla_{k(m)}$ are used as feedback to correct the data retrieved from the database (calibration database) during the next round of iterations. When m=n1, the data in the database (calibration database) has been calculated. Then this part can obtain outputs $\varepsilon_{k}$ and $\nabla_{k}$.
2. SINS navigation process: based on the SINS static data base (contains n2 sets of data), $\omega_{\mathrm{ib}(j)}^{b}$ and $f_{\mathrm{ib}(j)}^{b}$ can be used for SINS algorithm. Utilizing Equation (17) to calculate the square of position error:
  (17) $$\mathrm{RMS}E_{k}=\sqrt{\delta {\mathrm{Pos}}_{k(n2)}^{}}/n_{2}$$
  When j=n2, the process of inertial navigation comes to an end.

After the above process, the numerical function output $\mathrm{RMS}E_{k}$ can be obtained by the input $x_{k}$. Correspondingly we have derived the KF-based numerical function F(x). Bring F(x) into the algorithm flow chart shown in Figure 1. Let $x_{k}={\left[\begin{array}{cccccc} \varepsilon_{x} & \varepsilon_{y} & \varepsilon_{z} & \nabla_{x} & \nabla_{y} & \nabla_{z} \end{array}\right]}^{T}$, where $x_{k}$ is the IMU biases vector value. $F(x_{k})$. Afterwards, the minimum $\mathrm{RMS}E_{k}$ can be obtained by the AdaGrad algorithm, and the $x_{k}$ is the optimal value.
**Algorithm 2:** KF-based numerical function.**Input:** state vector $x_{k}={\left[\begin{array}{cccccc} \varepsilon_{x} & \varepsilon_{y} & \varepsilon_{z} & \nabla_{x} & \nabla_{y} & \nabla_{z} \end{array}\right]}^{T}$**Output:** position root mean square error ${\mathrm{RMSE}}_{k}$1. **loop 1 (Two position calibration process):** SINS algorithm:2. $C_{\mathrm{bk}}^{n}=C_{\mathrm{bk}-1}^{n}\left(I+T_{s}\omega_{\mathrm{nb}}^{b}\times \right)$3. $a_{k,k-1}^{n}=C_{\mathrm{bk}-1}^{n}f_{\mathrm{sfk}}^{b}-\left(2\omega_{\mathrm{iek}-1}^{n}+\omega_{\mathrm{enk}-1}^{n}\right)\times v_{k-1}^{n}+g^{n}$4. $v_{k}^{n}=v_{k}^{n}+T_{s}a_{k,k-1}^{n}$5. $Z_{k}=v_{k}^{n}-0$Kalman filtering:6. ${\hat{X}}_{k/k-1}=\Phi_{k/k-1}{\hat{X}}_{k-1}$7. $P_{k/k-1}=\Phi_{k/k-1}P_{k-1}\Phi_{k/k-1}^{T}+\Gamma_{k-1}Q_{k-1}\Gamma_{k-1}^{T}$8. $K_{k}=P_{k/k-1}H_{k}^{T}{\left(H_{k}P_{k/k-1}H_{k}^{T}+R_{k}\right)}^{-1}$9. $P_{k}=\left(I-K_{k}H_{k}\right)P_{k/k-1}$10. **until** calibration data base is used up.**return** $x_{k}$**end** loop 1 11. input $x_{k}$ into loop 2. 12. **loop 2 (SINS navigation process):**SINS navigation process:13. $C_{\mathrm{bk}}^{n}=C_{\mathrm{bk}-1}^{n}\left(I+T_{s}\omega_{\mathrm{nb}}^{b}\times \right)$14. $a_{k,k-1}^{n}=C_{\mathrm{bk}-1}^{n}f_{\mathrm{sfk}}^{b}-\left(2\omega_{\mathrm{iek}-1}^{n}+\omega_{\mathrm{enk}-1}^{n}\right)\times v_{k-1}^{n}+g^{n}$15. $v_{k}^{n}=v_{k}^{n}+T_{s}a_{k,k-1}^{n}$16. $L_{k}=L_{k-1}+\frac{T_{s}{\tilde{v}}_{\mathrm{Nk}-1}^{n}}{R_{M}+h_{k-1}}$$\lambda_{k}=\lambda_{k-1}+\frac{T_{s}{\tilde{v}}_{\mathrm{Ek}-1}^{n}{\mathrm{secL}}_{k-1}}{R_{N}+h_{k-1}}$$h_{k}=h_{k-1}+T_{s}{\tilde{v}}_{\mathrm{Uk}-1}^{n}$17. calculate $\delta \mathrm{po}s_{k}$18. **until** SINS static data base is used up **return** ${\mathrm{RMSE}}_{k}$**end** loop 2

## 4. Analysis of Experimental Tests

For the purpose of verifying the feasibility and evaluating the calibration accuracy of the KF-based AdaGrad algorithm. Both turntable rotation tests and field tests are performed with the KF-based AdaGrad algorithm. The rotation tests were carried out on the three-axis turntable. The sampling rate of the SINS is 4000 Hz, and the ring laser gyros are with an accuracy of $0.01^{\circ }/$h (1σ) and accelerometers are with 50 g (1σ).

**Figure 3:**
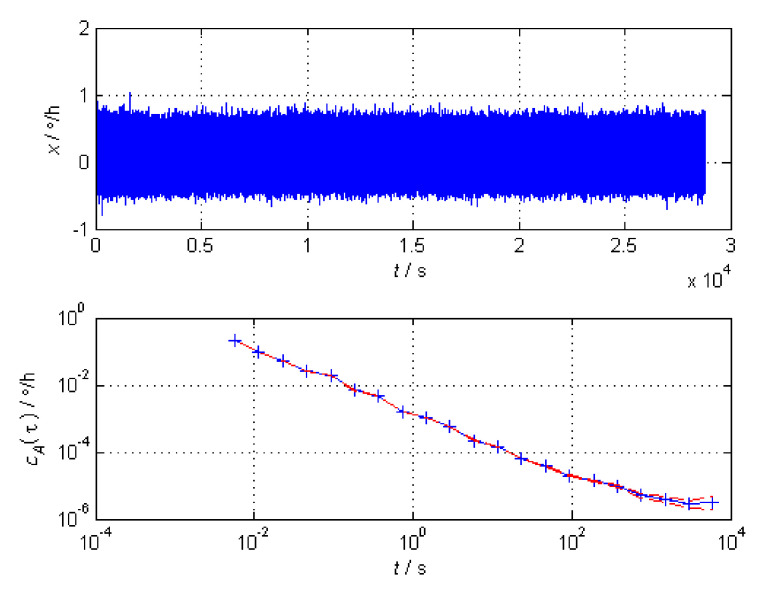
Gyroscope signal analysis.

The gyro signal (lasting 8 h) analysis utilizing Allan variance is shown in Figure 3.

As is shown in Figure 3, the three elements of Allan variance are quantization noise, angle random walk and bias instability. While KF is based on the hypothesis of GWN, the gyros we use usually have irregular noise.

For the purpose of analyzing the influence of the IMU biases on navigation errors. We list two tables to represent the error propagation including long-term navigation and short-term navigation. The long-term navigation error propagation is shown in Table 1 and the short-term navigation error is shown in Table 2.

As are shown in Table 1 and Table 2, the IMU biases would cause navigation error both in long-term navigation and short-term navigation. In order to verify the calibration effect, we need to carry out long-term navigation and short-term navigation experiments.

Through the comparison between Table 1 and Table 2. We can find out that in the short-term navigation, the gyro bias of the upward gyro does not affect the navigation accuracy in static condition. The position error of the long-term navigation process is affected by many error sources. Therefore, it is difficult to decompose the influence of IMU errors using conventional decoupling method.

Three car-mounted field tests were carried out in Xi’an, Shaanxi province and Chongqing to verify the effectiveness of the calibration methods. The car-mounted field tests in Xi’an are short-term experiments and the test in Chongqing is a long-term experiment. We utilized a DPGS with an accuracy of 3 cm as the reference position information. Compared with line search and two-position methods, the experimental results can prove the feasibility of the calibration method [32,33,34,35].

### 4.1. Static Tests

According to Section 3, this paper introduces two improved methods for gradient descent algorithm. In order to obtain the minimum value of the KF-based numerical function $F(x_{k})$ in a stable way, an algorithm performance analysis among these algorithms is needed.

**Figure 4:**
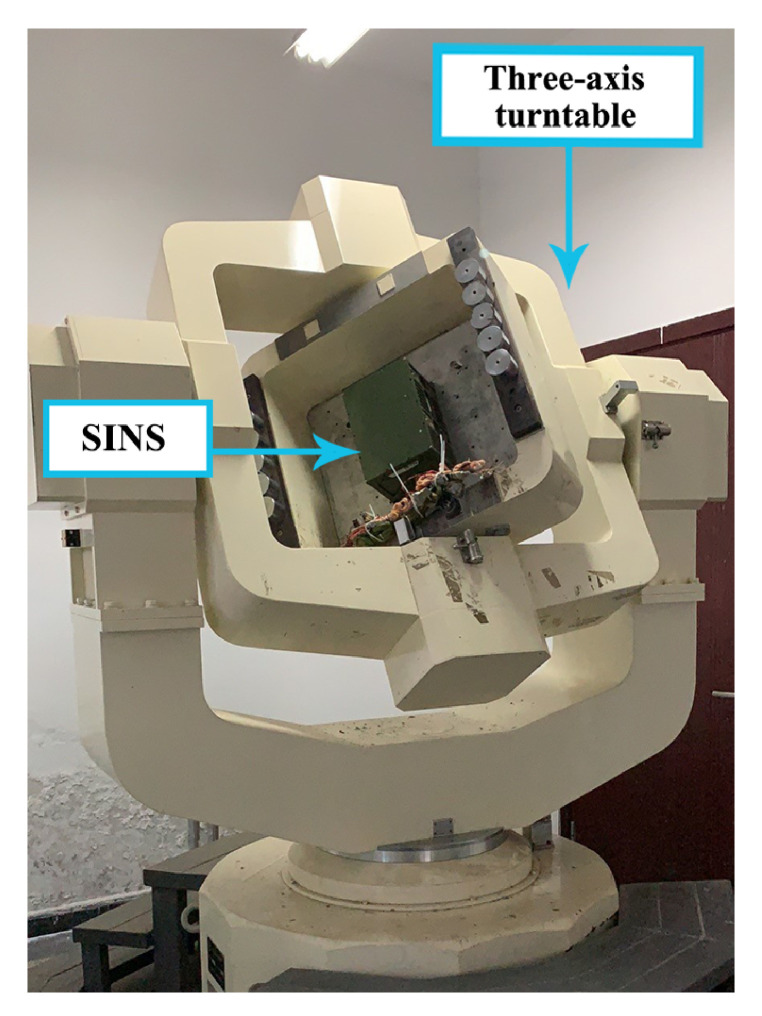
Calibration facilities.

In this subsection, static tests were utilized to obtain $F(x_{k})$. As is shown in Figure 4, SINS is stiffly fixed on a three-axis turntable. After confirming the fixation, calibration is started. The recorded data were saved as a calibration database and SINS static database. The flowchart of AdaGrad and the diagram of KF-based numerical function are also displayed in this paper. Static tests and calibration processes were carried out in a three-axis turntable.

The raw data including three gyros and three accelerometers from the experiment was collected by data collection computer. The collected data was utilized as the calibration database. Afterward, the static tests were carried out for 1 h, the IMU raw data was collected as SINS static database to build the numerical function $F(x_{k})$. We utilized the numerical function to compare line search and AdaGrad. Set $x_{k}$ equals zero vector as the initial value and $\mathrm{RMS}E_{k}$ as the function result. The number of iterations was set as 5000 because too many iterations are too much burden for a normal experiment computer. Results are shown in Table 3.

**Figure 5:**
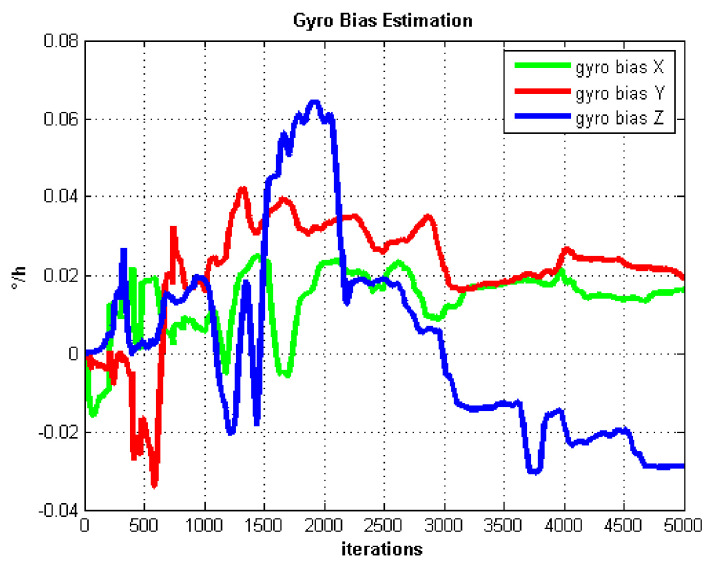
Gyro bias estimation.

**Figure 6:**
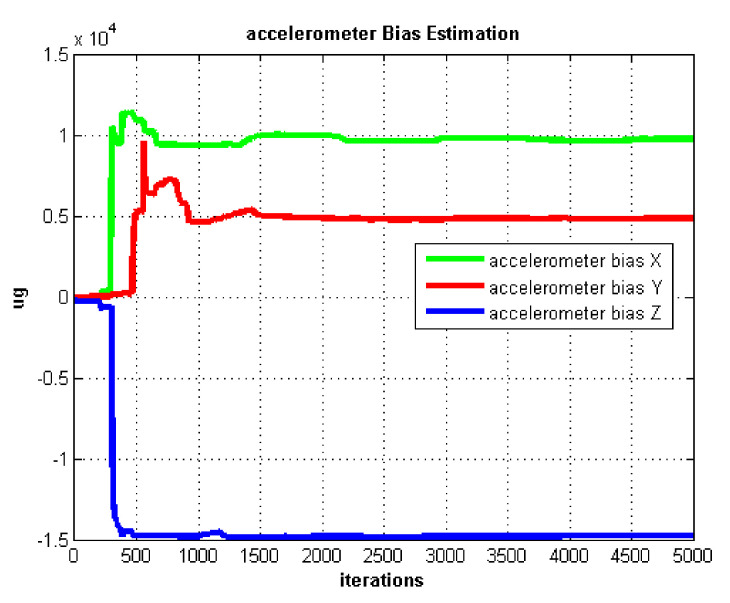
Accelerometer bias estimation.

The IMU biases estimation curves utilizing AdaGrad are shown in Figure 5 and Figure 6.

The self-rotation angular rate we set in the static test is 20 ${}^{\circ }$/s. In Figure 5, we can see the gyro biases converge slowly. Gyro biases converge after about 4000 iterations. In contrast, the accelerometer biases converge very fast after about 1000 iterations which are shown in Figure 6.

**Figure 7:**
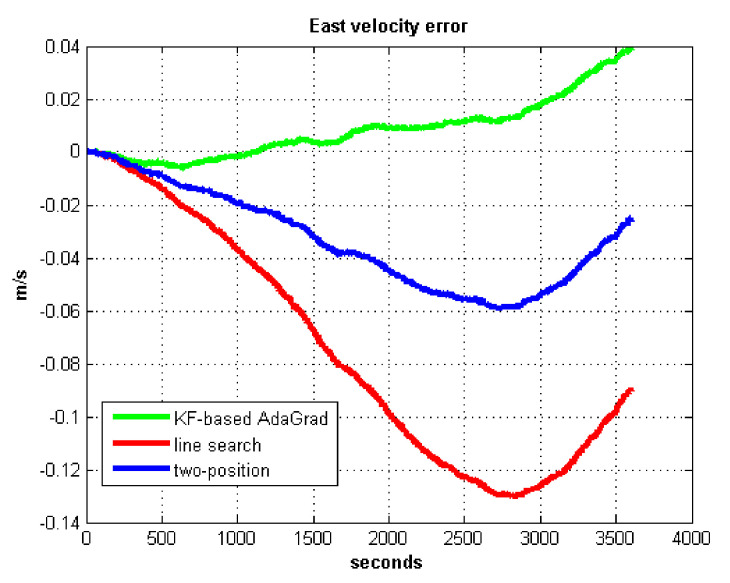
East velocity error.

**Figure 8:**
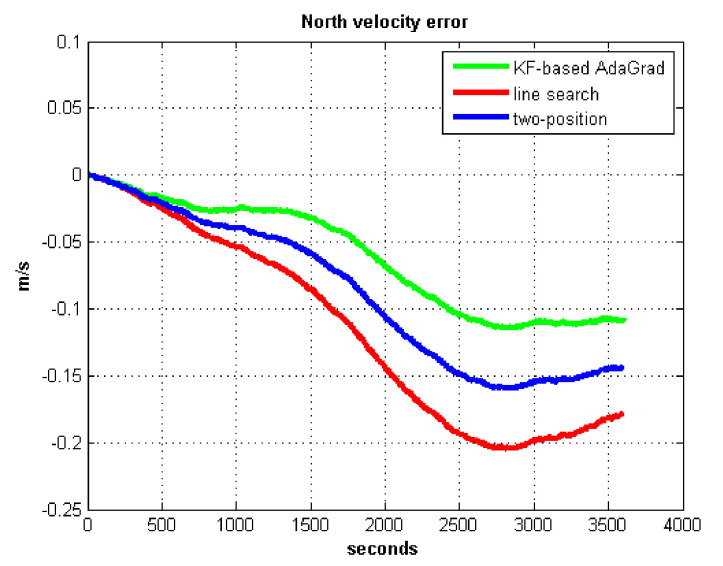
North velocity error.

**Figure 9:**
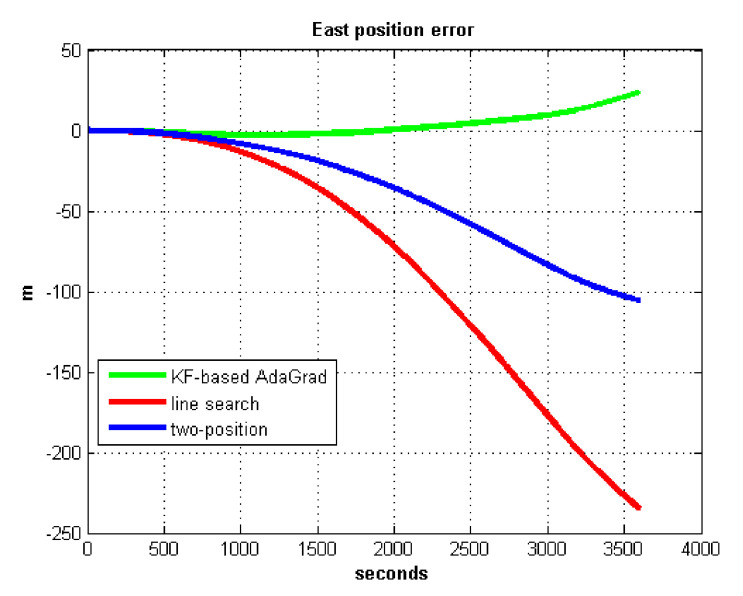
East position error.

**Figure 10:**
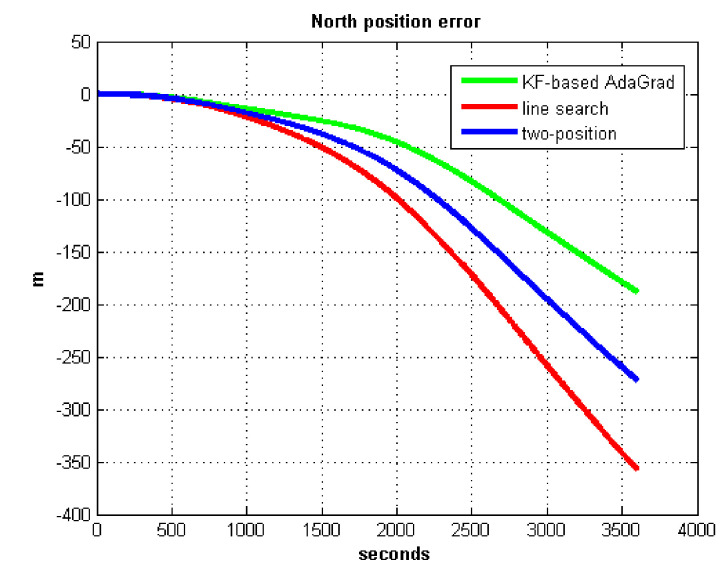
North position error.

The static test (lasts 1 h) experimental results are shown in Figure 7, Figure 8, Figure 9 and Figure 10.

**Figure 11:**
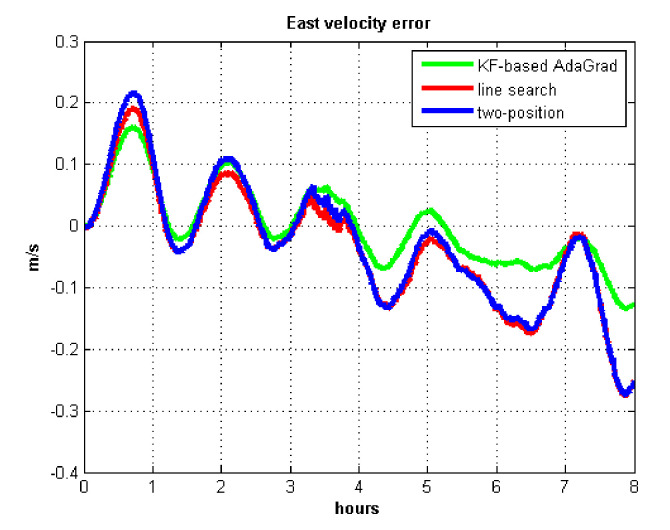
East velocity error.

**Figure 12:**
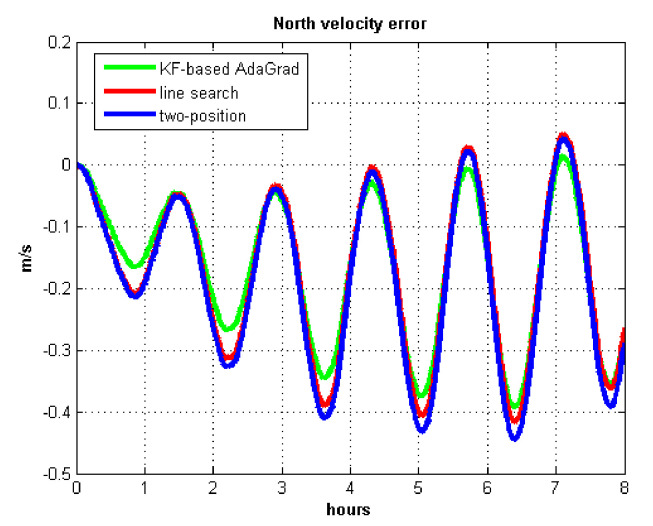
North velocity error.

**Figure 13:**
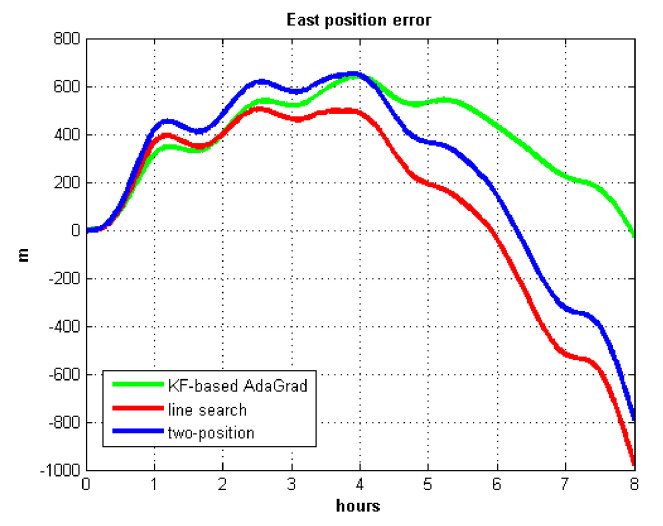
East position error.

**Figure 14:**
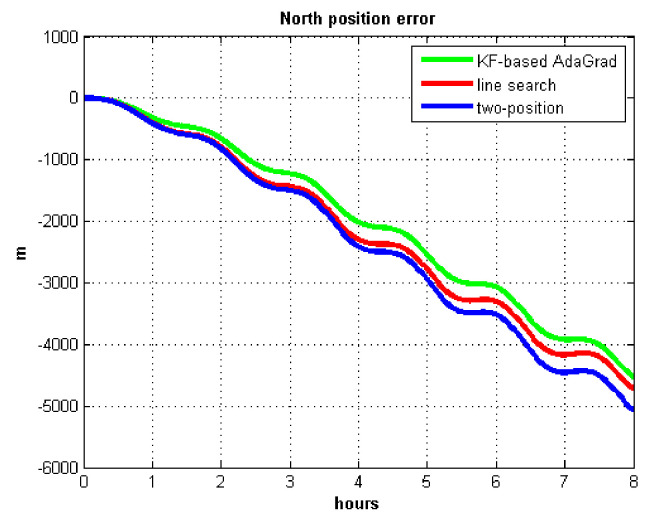
North position error.

We do not compare with the gradient descent method because in Table 3, the gradient descent method clearly entered into a locally optimal solution. Experimental results show that the convergence speed of line search is slower than AdaGrad. Due to the limit of iterations, the accuracy of the line search is lower than the AdaGrad method. The velocity and position errors show that the two-position calibration method is more accurate than the line search. AdaGrad has faster convergence rate and higher accuracy in static tests. In order to fully verify the calibration results in dynamic environment, 8 h static tests were carried out. The experimental results were shown in Figure 11, Figure 12, Figure 13 and Figure 14.

The velocity and position errors show that KF-based AdaGrad calibration method is more accurate than the line search and the two-position calibration method. The 8 h test shows the peak value of each north and east position errors. AdaGrad has higher accuracy in static test.

### 4.2. Field Tests

**Figure 15:**
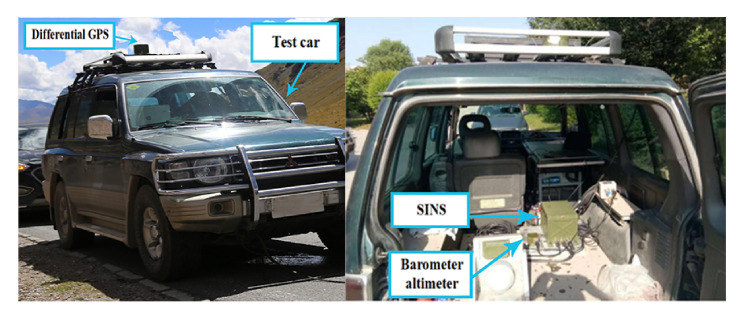
Experimental car and equipment.

The static test only considers the angular motion. In order to fully verify the proposed method in this paper, we need to consider the case with linear motion. The field tests were conducted in this subsection. As is shown in Figure 15, the SINS and the DGPS are equipped on the experimental car. The scale factors and installation angles were accurately calibrated before the field tests were carried out.

**Figure 16:**
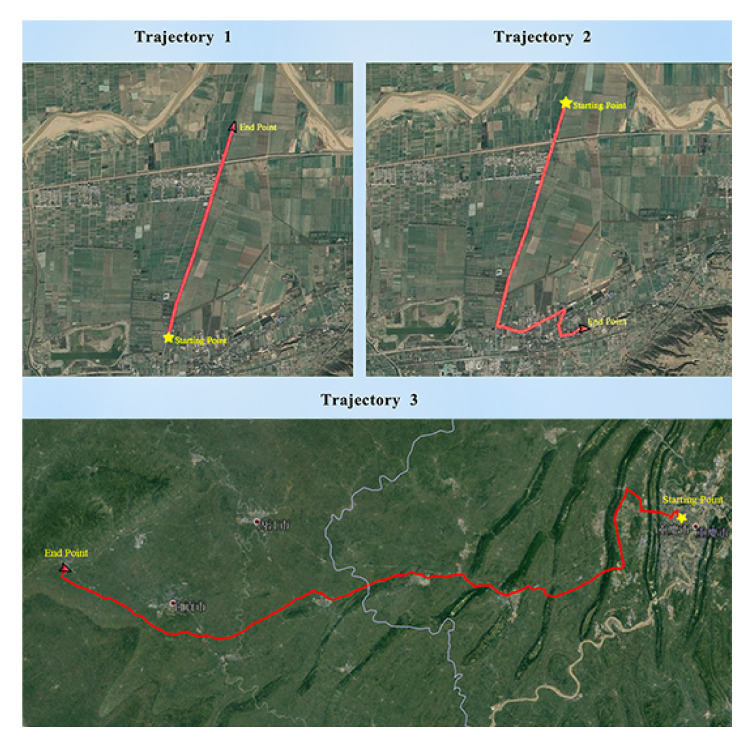
Field test trajectories.

Three field tests were carried out and the corresponding trajectories are shown in Figure 16. Table 4 shows the field test arrangements. During the maneuvering process of the vehicle, we utilize the SINS/GPS integrated algorithm to provide precise velocity and position information as the velocity and position reference. In order to compare the two-position algorithm, line search and AdaGrad, we utilize the same trajectories to compare the three algorithms.

Field test 1 is a straight-line road test and field test 2 contained several turns and one sharp turn. The test vehicle had no obstruction on the test roads thus the GPS signal is consistent and effective thus the SINS/DGPS integrated algorithm can provide stable position information and velocity information.

**Figure 17:**
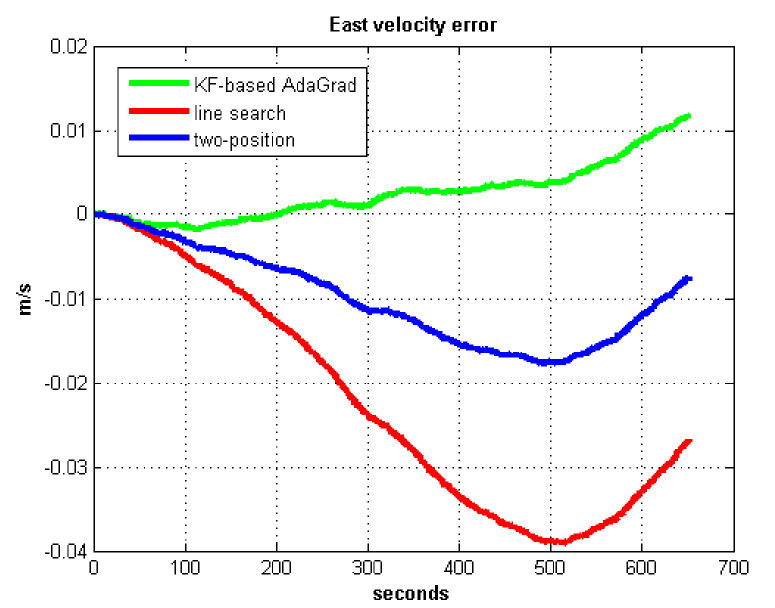
East velocity error in test 1.

**Figure 18:**
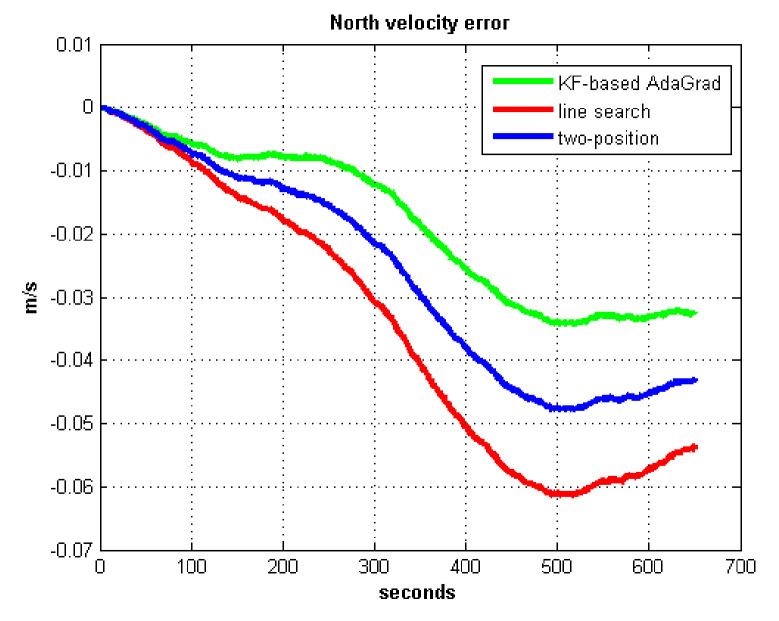
North velocity error in test 1.

**Figure 19:**
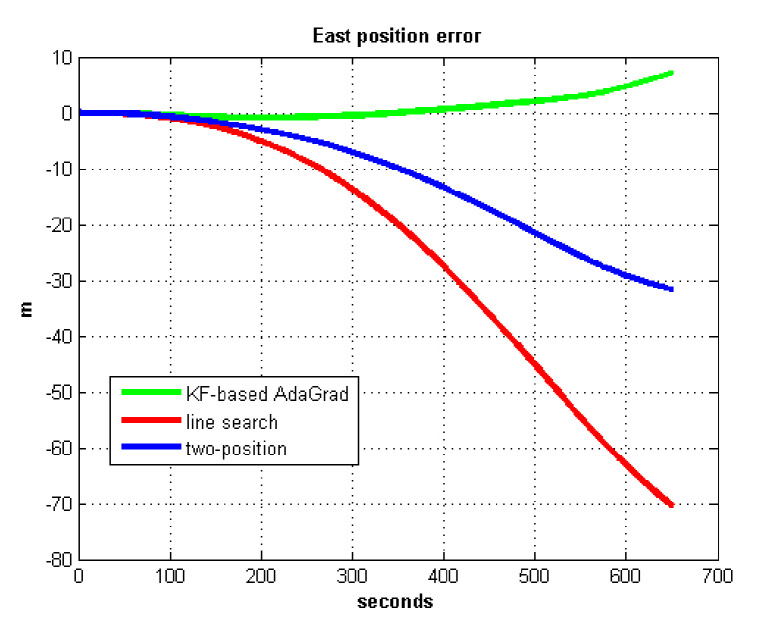
East position error in test 1.

**Figure 20:**
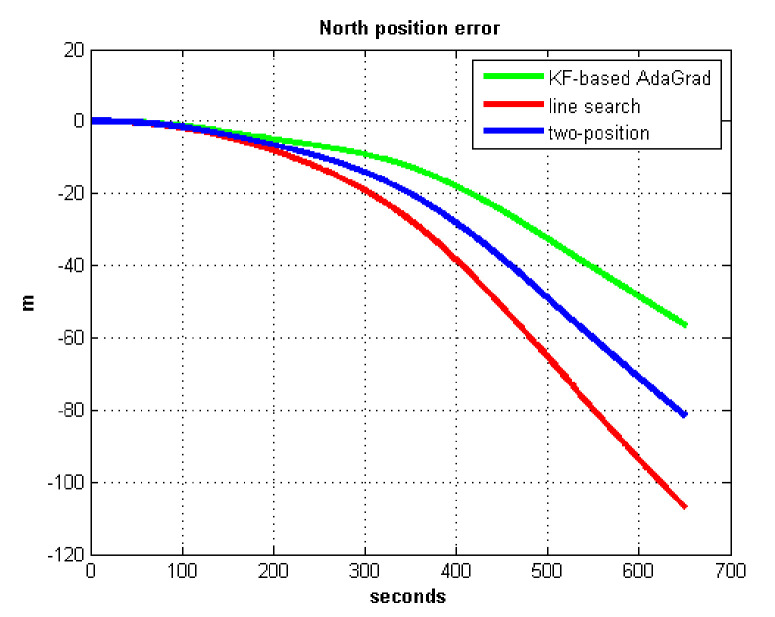
North position error in test 1.

The results of field test 1 are shown in Figure 17, Figure 18, Figure 19 and Figure 20.

We list the RMS and peak value of velocity and position error of test 1 in Table 5 and Table 6.

In field test 1, Figure 17 and Figure 18 shows that the east and north velocity errors of which the utilized KF-based AdaGrad are the smallest among the three methods. The two-position method is more accurate than the line search method. The position error is similar to the velocity error. Figure 19 and Figure 20 show that the east and north position error which utilized the two-position method smaller than the error of which utilized line search. The method utilized KF-based AdaGrad has the smallest position error.

**Figure 21:**
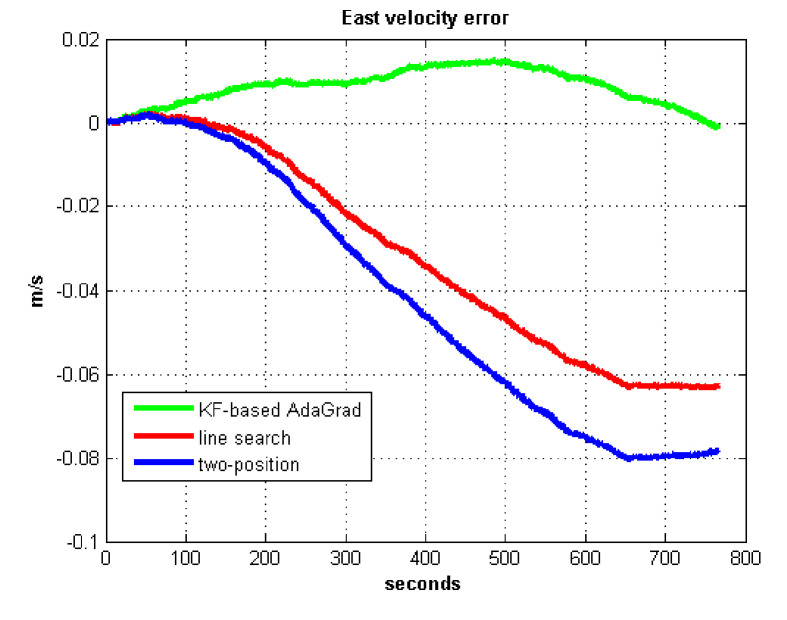
East velocity error in test 2.

**Figure 22:**
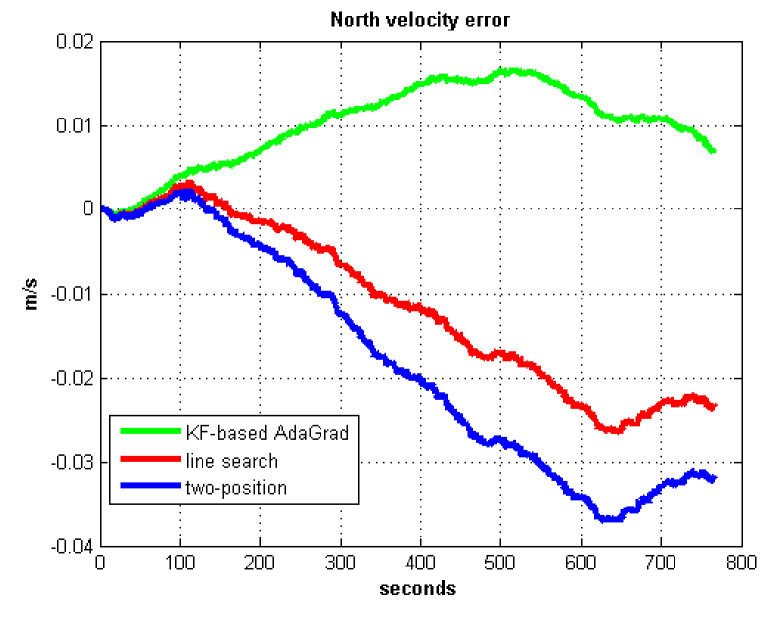
North velocity error in test 2.

**Figure 23:**
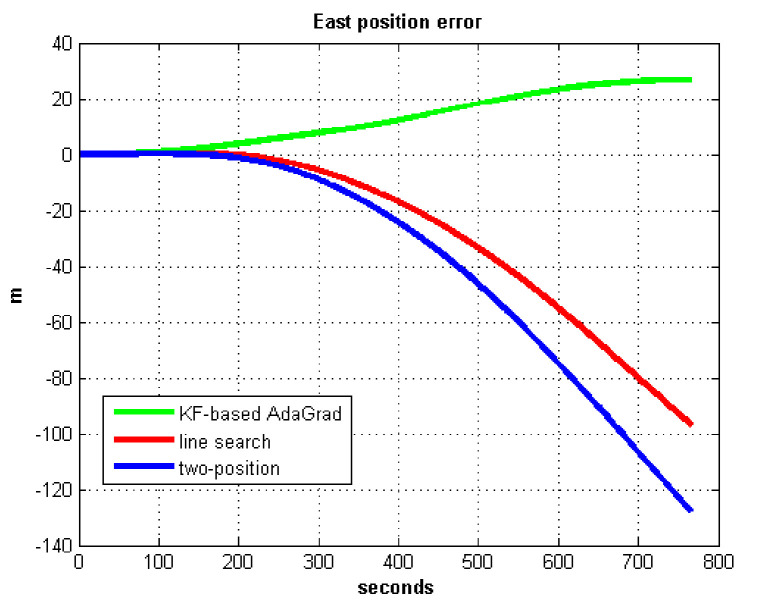
East position error in test 2.

**Figure 24:**
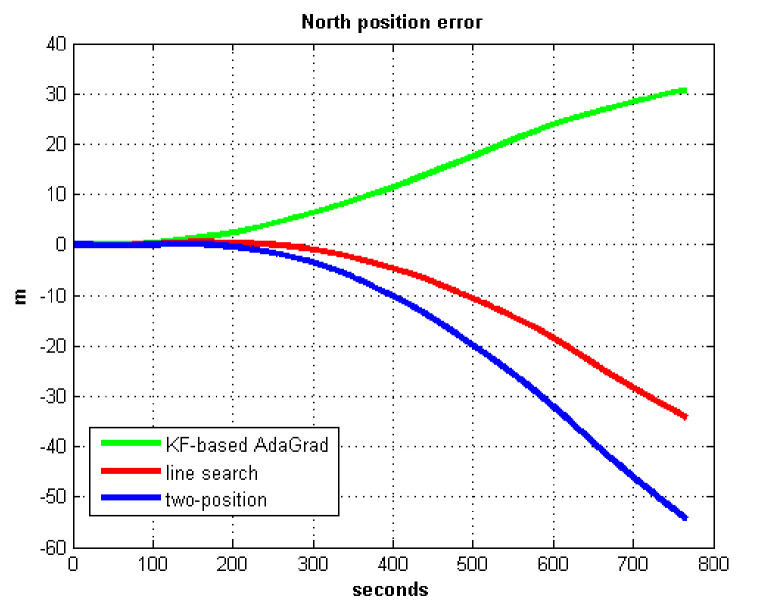
North position error in test 2.

The results of field test 2 are shown in Figure 21, Figure 22, Figure 23 and Figure 24.

In field test 2, Figure 21 and Figure 22 show that the east and north velocity error utilizing KF-based AdaGrad is the smallest among the three methods. The line search method is more accurate than the two-position method. The position errors are shown in Figure 23 and Figure 24, the east and north position error which utilized KF-based AdaGrad are obviously smaller than the other two methods. The position error of which utilized line search is smaller than the error of which utilized two-position method. The results of RMS and maximum values of velocity and position error are shown in Table 7 and Table 8.

For the purpose of fully verifying the effectiveness of the KF-based AdaGrad calibration method in the long-term navigation process, an 8 h field test was carried out in Chongqing to excite all three parts with different periods and amplitude. Due to the unstable GPS signal in field test 3, we only compare position errors because of the low accuracy of GPS velocity. The 8 h position error curve can fully verify the estimated accuracy of IMU biases.

**Figure 25:**
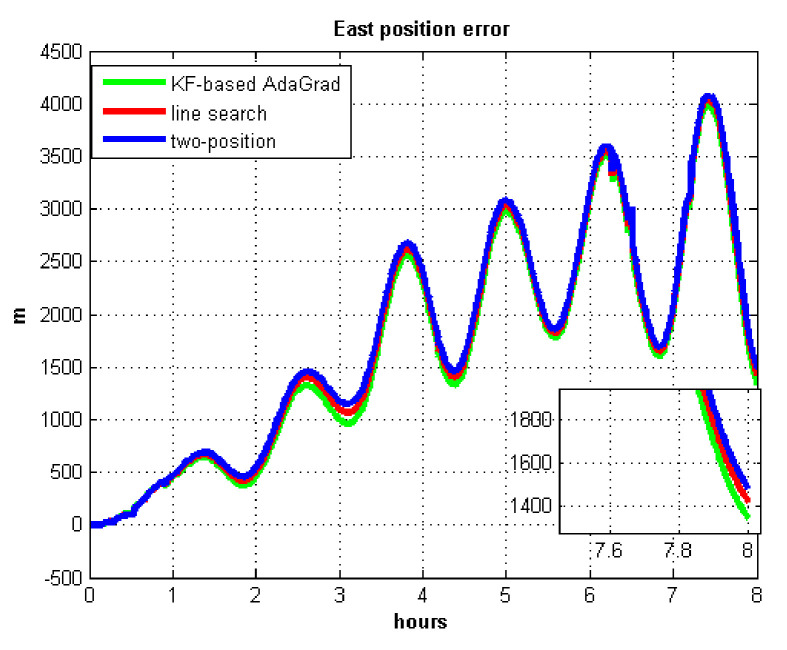
East position error in test 3.

**Figure 26:**
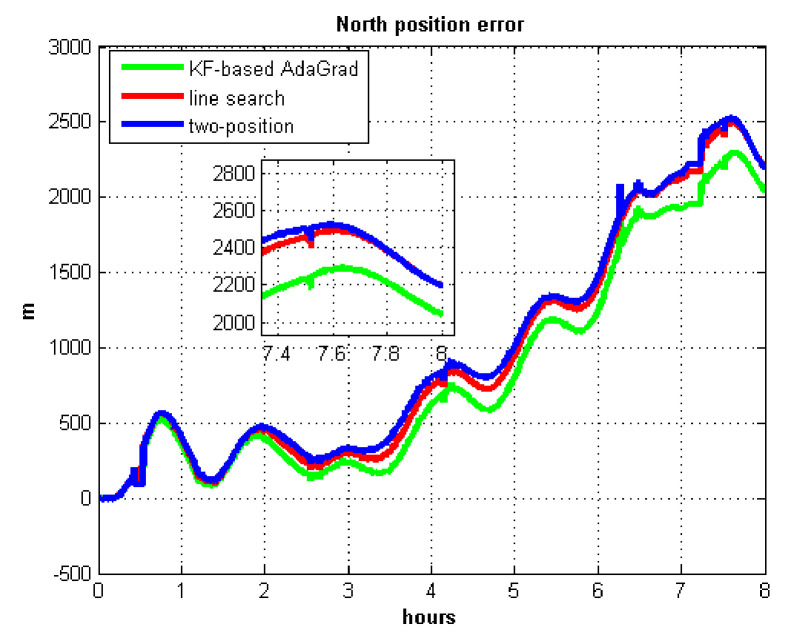
North position error in test 3.

The results of field test 3 are shown in Figure 25 and Figure 26.

In field test 3, the east and north position errors that utilized KF-based AdaGrad are smaller than the other two methods. Under long maneuvering condition, the line search and two-position calibration method have similar estimation accuracy.

The peak value of Figure 25 and Figure 26 is easy to figure out, we need to analyze the RMS of the position error. Table 9 shows the RMS of position errors in field test 3.

The results differences between field test 1 and field test 2 are the line search and two-position method because test 2 is more dynamic than test 1. Navigation errors due to IMU biases can be excited out more easily so the convergence rate of line search is faster in test 2. The AdaGrad has the highest accuracy among the three methods. In conclusion, under the severe maneuvering condition line search has higher accuracy than two-position method while under low maneuvering conditions the traditional method two-position is more accurate than line search. Both static tests and car-mounted tests show the proposed method can achieve a more accurate estimated velocity and position than the other two methods, which denotes that the KF-based AdaGrad can estimate the IMU biases more accurately. The convergence rate and accuracy of line search are greatly affected by the external dynamic condition. Thus, the line search is not fittable in IMU biases calibration.

## 5. Conclusions

The characteristics of IMU are usually difficult to determine. The IMU biases severely affecting navigation accuracy. In order to solve this problem, in this paper, we propose a KF-based AdaGrad calibration algorithm. We derive a KF-based AdaGrad numerical function and utilized AdaGrad method to solve the problem of setting step size. The recorded data were saved as the calibration database and the SINS static database. The flowchart of AdaGrad and the diagram of KF-based numerical function are also displayed in this paper. Static tests and calibration process were carried out in a three-axis turntable and three field tests were carried out in Xi’an. Experimental results show that the proposed calibration method can effectively improve the accuracy of estimation of IMU biases in both static and dynamic conditions.

## Figures and Tables

**Table 1:** Long-term navigation error propagation.

-	$\nabla_{E}$	$\nabla_{N}$	$\varepsilon_{E}$	$\varepsilon_{N}$	$\varepsilon_{U}$
δL	$\frac{\nabla_{E}}{g}c_{s}s_{f}$	$\frac{\nabla_{N}}{g}\left(1-c_{s}c_{f}\right)$	$-\varepsilon_{E}\left(\frac{s_{e}}{\omega_{\mathrm{ie}}}-\frac{s_{s}c_{f}}{\omega_{s}}\right)$	$-\varepsilon_{N}\left[\frac{s_{L}}{\omega_{\mathrm{ie}}}\left(1-c_{e}\right)-\frac{s_{s}s_{f}}{\omega_{s}}\right]$	$\frac{\varepsilon_{U}}{\omega_{\mathrm{ie}}}c_{L}\left(1-c_{e}\right)$
δλ	$\frac{\nabla_{E}}{g}e_{L}\left(1-c_{s}c_{f}\right)$	$-\frac{\nabla_{N}}{g}e_{L}c_{s}s_{f}$	$-\varepsilon_{E}e_{L}\left[\frac{s_{L}}{\omega_{\mathrm{ie}}}\left(1-c_{e}\right)-\frac{s_{s}s_{f}}{\omega_{s}}\right]$	$\varepsilon_{N}\left(c_{L}t+\frac{s_{L}t_{L}}{\omega_{\mathrm{ie}}}s_{e}-\frac{e_{L}}{\omega_{s}}s_{s}c_{f}\right)$	$\varepsilon_{U}s_{L}\left(t-\frac{1}{\omega_{\mathrm{ie}}}s_{e}\right)$

**Table 2:** Short-term navigation error propagation.

-	$\nabla_{E}$	$\nabla_{N}$	$\varepsilon_{E}$	$\varepsilon_{N}$	$\varepsilon_{U}$
δL	0	$\nabla_{U}\frac{t^{2}}{2R}$	$-\varepsilon_{E}\frac{gt^{3}}{6R}$	0	0
δλ	$\nabla_{E}\frac{t^{2}}{2Rc_{L}}$	0	0	$\varepsilon_{N}\frac{gt^{3}}{6Rc_{L}}$	0

**Table 3:** Position RMSE results.

Iterations	Gradient Descent	Line Search	AdaGrad
0	214.2765 m	214.2765 m	214.2765 m
10	150.2434 m	145.7865 m	134.9809 m
100	135.7649 m	108.9873 m	90.091 m
200	133.2765 m	87.9087 m	78.9007 m
1000	132.2653 m	76.9876 m	22.1107 m
2000	132.2653 m	72.8974 m	22.1098 m
5000	132.2652 m	70.7477 m	22.1097 m

**Table 4:** The arrangements of the field tests.

Field Test Number	Static Stage Time	Maneuvering Stage Time	Total Distance	Maximum Speed
Trajectory 1	300 s	651 s	3560.52 m	14.72 m/s
Trajectory 2	300 s	768 s	5870.08 m	16.65 m/s
Trajectory 3	900 s	8 h	267.98 km	30.65 m/s

**Table 5:** RMS of velocity and position errors.

-	Line Search	Two-Position	KF-Based AdaGrad
$\delta v_{E}$	0.005982 m/s	0.002265 m/s	0.001276 m/s
$\delta v_{N}$	0.01055 m/s	0.008769 m/s	0.005989 m/s
$\delta p_{E}$	26.6124 m	11.4123 m	5.1241 m
$\delta p_{N}$	32.9655 m	27.5421 m	21.4231 m

**Table 6:** Maximum errors of velocity and position.

-	Line Search	Two-Position	KF-Based AdaGrad
$\delta v_{E}$	−0.0389 m/s	−0.0178 m/s	0.0132 m/s
$\delta v_{N}$	−0.0613 m/s	−0.0489 m/s	−0.0311 m/s
$\delta p_{E}$	−71.6436 m	−31.2141 m	8.7231 m
$\delta p_{N}$	−108.0921 m	−81.9215 m	−58.9249 m

**Table 7:** RMS of velocity and position errors.

-	Line Search	Two-Position	KF-Based AdaGrad
$\delta v_{E}$	0.03982 m/s	0.04123 m/s	0.01076 m/s
$\delta v_{N}$	0.01255 m/s	0.01476 m/s	0.010989 m/s
$\delta p_{E}$	35.2412 m	38.5124 m	11.5613 m
$\delta p_{N}$	16.6278 m	21.0138 m	13.4163 m

**Table 8:** Maximum errors of velocity and position.

-	Line Search	Two-Position	KF-Based AdaGrad
$\delta v_{E}$	−0.0613 m/s	−0.0809 m/s	0.0169 m/s
$\delta v_{N}$	−0.0378 m/s	−0.0276 m/s	0.0165 m/s
$\delta p_{E}$	−127.2532 m	−97.5235 m	23.9873 m
$\delta p_{N}$	−33.8349 m	−53.6821 m	31.3452 m

**Table 9:** RMS of position errors.

-	Line Search	Two-Position	KF-Based AdaGrad
$\delta p_{E}$	1789.5758 m	1745.7639 m	1714.8437 m
$\delta p_{N}$	987.0832 m	956.8051 m	914.5033 m
